# Chronic low back pain clinical outcomes present higher associations with the STarT Back Screening Tool than with physiologic measures: a 12-month cohort study

**DOI:** 10.1186/s12891-015-0669-0

**Published:** 2015-08-19

**Authors:** Isabelle Pagé, Jacques Abboud, Julie O᾽Shaughnessy, Louis Laurencelle, Martin Descarreaux

**Affiliations:** Département des sciences de l’activité physique, Université du Québec à Trois-Rivières (UQTR), Trois-Rivières, Québec Canada; Département d’anatomie, UQTR, Trois-Rivières, Québec Canada; Département de chiropratique, UQTR, Trois-Rivières, Québec Canada; Département des sciences de l’activité physique, UQTR, Trois-Rivières, Québec Canada; Département des sciences de l’activité physique, Université du Québec à Trois-Rivières, 3351 Boul. Des Forges, Trois-Rivières, G9A 5H7 Québec Canada

## Abstract

**Background:**

Stratification strategies based on identifying patient’s prognosis in order to guide patient care constitute one of the most prominent and recent approach in low back pain research. The STarT Back Screening Tool (SBST) although promising, has not been studied in patients with chronic low back pain (cLBP). Considering how challenging it is to translate research into practice, the value of integrating a new tool should be thoroughly assessed. The purpose was therefore to assess associations between the short- and long-terms clinical status and two types of variables, physiologic measures and the SBST, in participants with cLBP. The ability of both types of variables to discriminate between participants with and without higher levels of disability, pain, fear of movement and patient’s global impression of change was also investigated.

**Methods:**

Fifty-three volunteers with cLBP participated in an initial evaluation and follow-ups at 2-, 4-, 6- and 12-month. Physiologic measures (maximal voluntary contraction, maximal endurance and muscle activity evaluated during prone and lateral isometric tasks) and the SBST were assessed at baseline. Disability (Oswestry Disability Index, ODI), pain intensity (101-point Numerical Rating Scale, NRS), fear of movement (Tampa Scale for Kinesiophobia, TSK) and patient’s global impression of change (7-point scale, PGIC) were evaluated at baseline and at each follow-up. Aside the use of correlation analyses to assess potential associations; ROC curves were performed to evaluate the discriminative ability of physiologic measures and the SBST.

**Results:**

The SBST allowed for the identification of participants presenting higher levels of disability (ODI ≥24 %), pain (NRS ≥37 %) or fear of movement (TSK ≥41/68) over a 12-month period (AUC = 0.71 to 0.84, *p*s < 0.05). The SBST score was also correlated with disability at each follow-up (τ = 0.22 to 0.33, *p*s < 0.05) and with pain intensity and fear of movement at follow-ups. Among physiologic measures, only maximal voluntary contraction was correlated to disability, pain intensity or fear of movement during the follow-up (|τ| = 0.26 to 0.32, *p*s < 0.05) and none was able to identify participants presenting higher levels of outcomes (AUC *p*s > 0.05).

**Conclusion:**

Physiologic measures obtained during prone and lateral tests have limited associations with the clinical status over a 12-month period in patients with nonspecific chronic low back pain. On the other hand, the STarT Back Screening Tool is useful for the identification of patients who will present higher levels of disability, pain intensity and fear of movement over a year.

**Trial registration:**

Clinicaltrials.gov NCT02226692

## Background

Low back pain (LBP) constitutes a major public health issue as more than 85 % of patients who suffer from it are diagnosed with LBP of nonspecific origin [1]. The 2010 Global Burden of Disease Study reported LBP as the leading musculoskeletal cause of years lived with disability [2]. Moreover, a recent systematic review on the natural history of LBP concluded that this condition is relatively stable over time, and that becoming pain free should be considered an exception rather than the norm [3]. Considering the high prevalence of this condition, the heterogeneous patient presentations and the similar pattern of improvement characterizing a wide range of primary care treatments, stratified care has been identified as a research priority within the LBP field [4–6]. Foster et al. [5] described stratified care as a “strategy involving the identification of patient subgroups based on key characteristics such as their prognostic profile, likely response to specific treatment and suspected underlying causal mechanisms”. In addition, Hingorani et al. [7] mentioned that stratified care aims to optimize treatment, increase efficiency of healthcare and reduce unnecessary harm.

One of the most prominent and recent approach deals with targeting treatment to patient subgroups based on patients prognosis [5]. Although some prognostic indicators have been associated with long-term disability regardless of the patient’s pain status (acute, subacute, or chronic LBP), it is generally accepted that prognostic indicators can vary according to nonspecific LBP duration [8]. Indeed, the natural courses of acute, subacute and chronic LBP have been reported to differ [9]. A literature review exploring short- and long-term prognostic factors for pain intensity, disability, return to work and global perceived effect in patients with chronic nonspecific LBP has been recently published [10]. The authors reported that only lower pain intensity and lower physical job demands at baseline are associated with one of the reported outcomes (i.e. earlier return to work).

Considering the challenge of identifying prognostic factors, other research avenues emerged. Indeed, over the past years, several questionnaires identifying patients risk of persistent LBP and suggesting a stratified care have been developed [11, 12]. One of these questionnaires, the Subgroup for Targeted Treatment (STarT) Back Screening Tool (SBST), which allows patients categorization into three risk subgroups of persistent disabling LBP (low, medium, or high risk subgroup), has increasingly been studied since its validation in 2008 [13]. This tool has mostly been validated for patients with LBP of any duration in primary care setting (e.g. [14, 15]); however, different subgroups cut-offs might be observed in different populations, such as in secondary care settings [16]. On the other hand, trunk extension fitness (i.e. strength or endurance) and electromyography (EMG) power spectral parameters of the low back obtained during these exercises can predict first time LBP or symptoms recurrence and discriminate between patients with and without LBP [17–22]. Although heterogeneous results have been reported regarding associations between trunk muscle fitness and outcomes [23], trunk strength and endurance tests are commonly used by health professionals to initially evaluate patients with LBP, and exercises are often prescribed as part of rehabilitation [24]. Various tests have been proposed to evaluate the trunk extension fitness, but the Biering-Sørensen test (or the modified Biering-Sørensen test [25]), is considered as the gold standard [26]. The side-bridge test and its modified versions, is also advocated in the evaluation of patients with LBP since it results in lower compressive forces than other trunk exercises [27].

Bearing in mind that it remains a challenge to implement new research evidences into clinical practice [28] one can anticipate that clinicians will prefer stratified care approaches based on commonly used physical tests rather than a new and cost-effective tool such as the SBST [15]. Therefore, the aim of the present study was to assess the associations between the short- (≤6 months) and long- (12 months) terms clinical status and two types of variables, physiologic measures and the SBST, in patients with chronic nonspecific LBP. As a second objective, the ability of both types of variables to discriminate between participants with and without higher levels of disability, pain, fear of movement and patient’s global impression of change was investigated. It was hypothesized that both physiologic measures and the SBST would be associated to the short- and long-term clinical status, but that, based on recent evidences, the SBST would present a higher discriminative ability than physiologic measures.

## Methods

### Participants

Participants were recruited among the university’s community and through an advertisement in the local newspaper. Volunteers were first screened by clinicians at the outpatients’ chiropractic clinic in order to assess for the various inclusion and exclusion criteria. The inclusion criteria were adults between 18 and 60 years old with nonspecific chronic LBP, able to read and understand French. Nonspecific LBP was defined as a pain located between the twelfth rib and the inferior gluteal fold for which no specific source of pain could be identified. “Chronic” was defined as a pain present for 12 weeks or more, and included both constant and recurrent patterns of pain. The exclusion criteria were defined prior to the recruitment effort; they included LBP of specific origin [1], spine surgery or trauma, scoliosis, neurological disease, uncontrolled hypertension, pregnancy, recent lumbar cortisone injection, being under medications known to impair physical effort and pain perception, active lower body injury and/or severe, pain irradiating below the knee [29], and disabling pain limiting the capacity to undergo the evaluation. Once included in the study, participants were contacted by the researcher to schedule for baseline assessment and to give their informed written consent according to the university’s Human Research Ethics Committee certification (No. CER-12-181-06.22).

### Experimental protocol

The baseline assessment (T0) was conducted at the university’s Neuromechanics and Motor Control Laboratory and lasted approximately 2 h. Participants were first asked to complete clinical questionnaires in order to assess lumbar disability (Oswestry Disability Index, ODI), actual pain intensity and mean pain intensity in the past 2 months (101-point Numerical Rating Scale, NRS), fear of movement (Tampa Scale for Kinesiophobia, TSK), and prognosis (SBST [13]). All these questionnaires have been reported to be reliable and responsive in the treatment of chronic LBP and their French versions, which were used, have been validated [30–33]. The SBST assesses the risk of poor prognosis through nine questions relating to comorbidity, disability, pain catastrophizing, fear of movement, bothersomeness, anxiety and depression symptoms [13]. A scoring system provides a risk group (low, medium, or high). Although a psychological subscale is measured by items 5 to 9, the present study only refers to the total score (range: 0 to 9).

Participants were then instructed to perform, in randomized sequences, isometric trunk muscle endurance tasks in prone, right lateral and left lateral positions, each preceded by a maximal isometric voluntary contraction (MVC) in the same position. The experimental tasks were thoroughly explained and demonstrated by the researcher before any data were recorded. Lumbar muscle activity was collected during prone endurance and MVC tasks using surface electromyography (EMG) matrices. Subsequently, participants were contacted for a follow-up at 2 months (T1), 4 months (T2), 6 months (T3), and 12 months (T4) by telephone, email or mail, at the participant’s convenience.

### MVC and endurance tasks

**Fig. 1:**
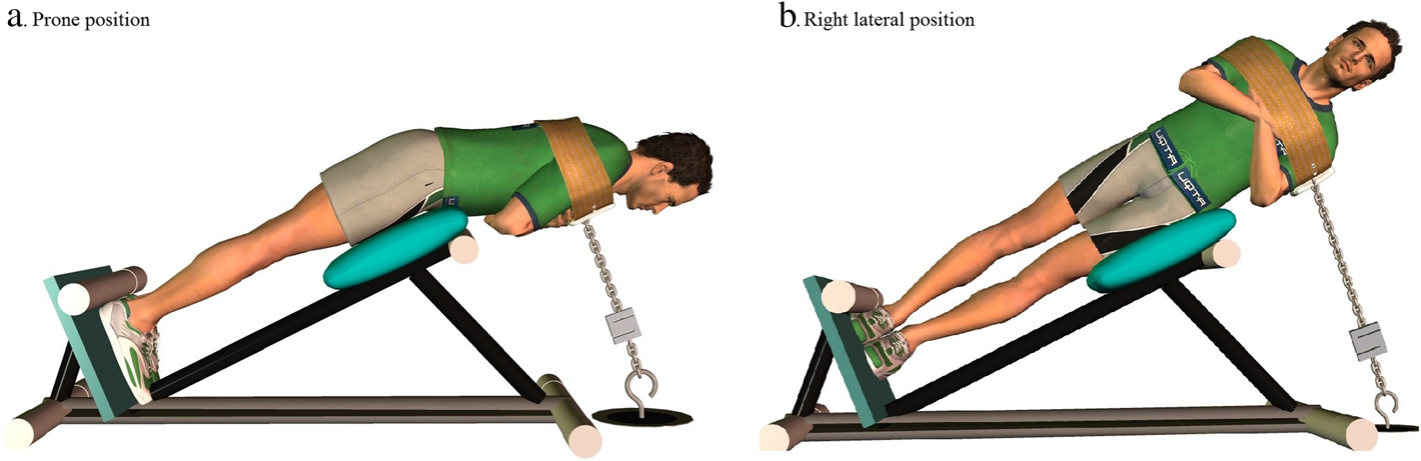
**a**. Prone position and **b**. Right lateral position. These positions were used to assess the endurance and the MVC of trunk muscles

Prone and lateral tasks (Fig. 1) were performed according to the protocols presented by Champagne et al. [25] and Pagé and Descarreaux [34]. Lateral position tasks were labelled according to the side up (i.e. when participants were lying on the left side, the task was called right lateral endurance or MVC task, as shown in Fig. 1b). Participants were positioned on a 30° Roman chair, straight upper body, the iliac crest aligned with the chair’s border and the arms crossed on the chest. A fixed harness, installed over their shoulders, was connected inline to a uni-axial force transducer on the floor (NTEP-87-057A3 class III, Artech, Riverside, CA, USA).

A MVC task (prone, right and left lateral) was performed just prior to the respective trunk endurance task by slowly extending the trunk until feeling a tension on the harness (straight body). At this point, the participants were asked to perform a maximal trunk exertion contraction against the harness for 5 s. A computer monitor was located in front of the participant in order to provide him a visual feedback of his/her performance. The first trial was performed without feedback, but during the subsequent trials the participants visualized a force threshold of 10 % superior to the previous trial. MVC was considered completed when the participants could not reach the threshold, or after completing three trials. During endurance tasks, the participants were asked to maintain, as long as they could, 30 % ± 5 % of their MVC carried out just before. A visual feedback was provided using a computer monitor throughout the task, in addition to verbal position correction provided by the researcher. The task was considered “completed” when the participants chose to stop or swayed too much from the initial position (as evaluated by the researcher) or target force. Two minutes of rest were allowed between MVC and trunk endurance tasks. Verbal encouragements were provided throughout endurance and MVC tasks.

For data analyses, an average lateral position MVC (variable defined as lateral MVC) and an average lateral position endurance time (variable defined as lateral endurance) were calculated by averaging the MVCs and endurance times obtained during right lateral position task and left lateral position task.

### EMG acquisition and analyses

**Fig. 2:**
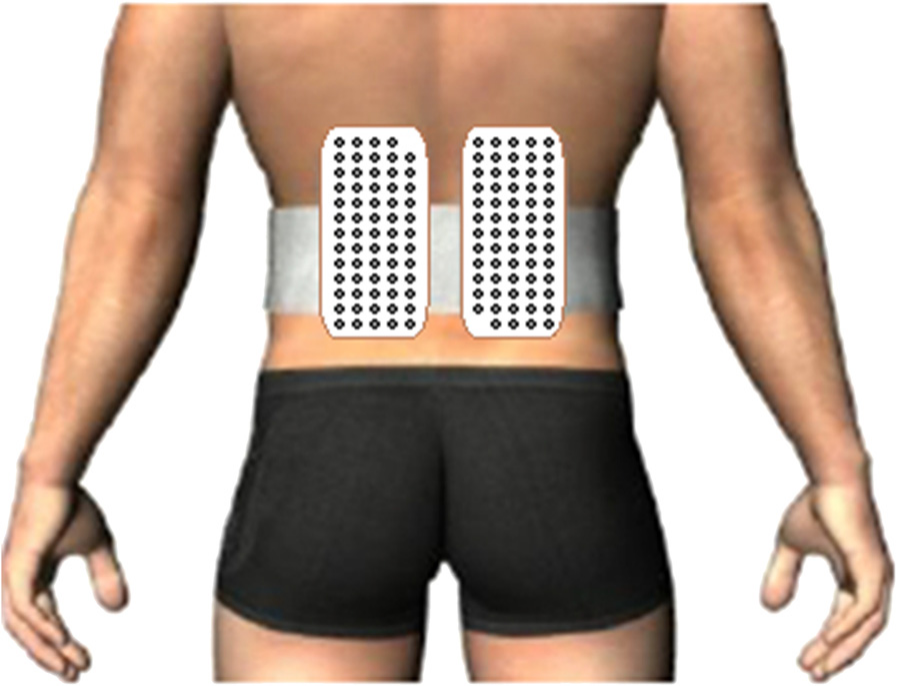
Matrices used in the recording of lumbar muscles activity during prone position tasks

EMG of right and left lumbar erector spinae was recorded during prone position tasks using two adhesive matrices of 64 electrodes (model ELSCH064; LISiN-OT Bioelettronica; Torino, Italy) as illustrated in Fig. 2. The array grid consisted of 64 electrodes, 13 rows × 5 columns (2 mm diameter, 12.5 mm inter-electrode distance). The electrode surfaces were separated from the skin by a small cavity (approximately 1 mm thick) filled with electrolyte gel (AC-CREAM250V; Spes Medica; Battipaglia, Italy). The center of each grid was located at L3 and two ground electrodes were placed on the right and left olecranon processes. Skin impedance was reduced by shaving body hair; gently abrading the skin with fine-grade sandpaper (Red Dot Trace Prep, 3 M; St. Paul, MN, USA), and wiping the skin with alcohol swabs. The bipolar EMG signals were amplified (64-channel surface EMG amplifier, SEA 64, LISiN-OT Bioelettronica; Torino, Italy; −3 dB bandwidth 10–500 Hz) by a factor of 5000, sampled at 2048 Hz, and converted to digital form by a 12-bit A/D converter. The data were collected using the OT Bioelettronica custom software and processed by Matlab (MathWorks; Natick, MA, USA).

Each bipolar EMG signal obtained from both matrices during isometric prone endurance task was digitally band-pass filtered in the frequency bandwidth 20–450 Hz (2^nd^ order Butterworth filter). Notch filters were also applied to reject 60 Hz power line interference and its harmonics. Each electrode filtered signal was then divided in windows of 0.5 s for which an individual root mean square (RMS) value was computed and normalized with the corresponding RMS obtained during prone MVC task. The center of gravity of each two-dimensional representation of RMS values was determined and the spatial migration of muscular activity throughout the endurance task was quantified. Global mean migration of muscular activity (variable defined as motor variability) was calculated by averaging both left and right sides mean migration for each participant.

Individual median frequency (MF) value was also calculated for each window of 0.5 s. MF was defined as the requency that divides the spectrum into two equal areas. Median frequency slope (MFslope) was obtained from the slope of a linear regression fit the MF values for each electrode. The MFslopes were then divided by the initial MF (iMF, t = 0) obtained from the interception of MFslope for each electrode and averaged for each side (NMFslope in %S^−1^) [35]. Global NMFslope (variable defined as NMFslope) was finally calculated by averaging both left and right sides mean NMFslope for each participant.

### Outcome assessments

At each follow-up assessment (T1, T2, T3 and T4), mean pain intensity since the last follow-up, lumbar disability, and fear of movement were re-evaluated in order to quantify participant clinical status. Furthermore, a 7-point patient’s global impression of change scale (PGIC) at T3 and T4 was used to measure participant’s level of perceived change in the past 6 months.

### Dichotomous outcomes

All outcome variables (ODI, NRS, TSK and PGIC) were re-coded into dichotomous outcomes at T3 and T4 based on Wideman et al. [36] and Hill et al. [37]. Consistent with these studies, presence of disability was defined as a follow-up score of ≥24 % on the ODI, presence of pain as a score of ≥37 % on the NRS, presence of fear of movement as a score of ≥41 on the TSK, and presence of subjective status change as a score of 1 or 2 (very much or much improved) on the PGIC.

### Approach to data analysis

Sample size was calculated using an estimated moderate effect size (0.40 ≤ r ≤ 0.50 [38]) with a significance level of 0.05, a desired power of 0.80 and an estimated attrition of 10 %. Given the aforementioned requirements, the number of participants needed was between 51 (*r* = 0.40) and 32 (*r* = 0.50). The t-test for two samples was used to compare baseline characteristics (age, body mass index, disability, fear of movement, pain intensity, SBST score, endurance times and MVCs) between males and females, and between participants and lost to follow-up.

To address our objectives, Kendall tau rank correlation coefficient was computed to assess the relationship between physiologic measures (endurance times, MVCs, motor variability and NMFslope) and the SBST, and clinical outcomes (NRS, ODI, TSK and PGIC) at T0, T1, T2, T3 and T4: Kendall’s technique was used for all pairs of variables, as most of them did not meet the continuity or normality requirements of Pearson’s correlation measure. The importance of the correlation was evaluated as being large (> 0.34), moderate (0.20–0.34) or small (< 0.19), using equivalence formula between Pearson correlation coefficient and Kendall tau rank correlation [39]. The area under the curve (AUC) statistic from receiver operating characteristic (ROC) curves and 95 % confidence intervals (CI_95_) were also used to describe the ability of the physiologic measures and the SBST to identify participants presenting disability, pain, fear of movement or an absence of subjective status change at T3 and T4. Ability strength was defined according to previous studies on the SBST : 0.7–0.8 indicated acceptable ability, 0.8–0.9 indicated excellent ability and 0.9 or over indicated outstanding ability [13]. Whenever AUC was significant, predictive validity was assessed by calculating sensitivity, specificity and positive and negative likelihood ratios (LRs) for different cut-offs values. It is generally accepted that a positive LR higher than 10 significantly increases the probability of the specific condition, while a negative LR lower than 0.1 significantly decreases the probability of the specific condition [40].

For all statistical analyses, *p* < 0.05 was considered to be statistically significant. Statistical analysis was performed using SPSS statistical package version 19.0.0.

## Results

### Study sample

**Fig. 3:**
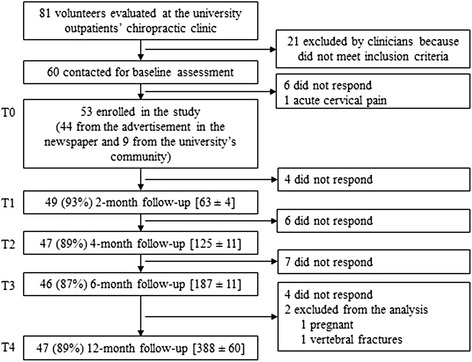
Flow of participants through the study. Within parenthesis are the percentages of total participants at baseline (T0). Within brackets are the means (± SD) of number of days for completion

The flow of participants through the study with reasons for exclusion and lost to follow-up is presented in Fig. 3. Overall, 53 volunteers - 30 males and 23 females with a mean age of 44.09 years (range: 21–60) - with nonspecific chronic LBP were included in the study. At least 87 % of the study sample completed each follow-up. Baseline characteristics of the sample are summarized in Table 1. No difference in baseline characteristics were identified between participants and lost to follow-up. Since no differences were found between genders either, all further statistical analyses were performed without taking gender into account.

**Table 1:** Participant’s baseline characteristics

Characteristic	Mean ± SD
Number (female : male)	23 : 30
Age (years)	44.09 ± 13.26 (range : 21 to 60)
Employment	
Unemployed : worker : retired	3 : 38 : 4
Housewife : student	1 :7
Weight (kg)	76.19 ± 15.27
Height (m)	1.70 ± 0.09
Body mass index (kg/m^2^)	26.14 ± 4.36
Duration of symptoms (months)	130.68 ± 112.03 (range : 4 to 360 months)
Reported physical activity by week (hours)	4.33 ± 3.27 (range : 0 to 14 h)
Initial disability (%)	18.38 ± 10.33
Initial fear of movement (/68)	36.42 ± 8.75
Initial pain intensity (%)	27.61 ± 22.94
Prone endurance (s)	57.46 ± 27.10
Lateral endurance (s)	31.73 ± 15.40
Prone MVC^a^ (N)	147.90 ± 64.58
Lateral MVC (N)	112.67 ± 49.93
SBST^b^ risk group (low : moderate : high)	35 : 11 : 7

^a^Maximal isometric voluntary contraction

^b^STarT Back Screening Tool

### Correlations between SBST and physiologic measures, and outcome variables

At baseline assessment, Kendall tau rank correlation coefficient analysis (see Table 2) showed that, few physiologic measures were correlated to disability or fear of movement (moderate or strong correlation) but any to pain intensity. On the other hand, the SBST presented a strong correlation with disability, and fear of movement. When correlation at follow-ups were evaluated, only prone and lateral MVC were significantly correlated to an outcome variables while the SBST was correlated to disability at each follow-up, to pain intensity at T4, and to fear of movement at T1. Interestingly, the SBST was not correlated to PGIC at neither T3 nor T4. Significant correlation coefficients are reported in Table 3.

**Table 2:** Correlation coefficients between both the SBST^a^ and physiologic measures, and outcome variables at baseline

Variable	Disability	Pain intensity	Fear of movement
Prone endurance (s)	−0.26^*^	−0.04	−0.22^*^
Lateral endurance (s)	−0.11	0.07	−0.17
Prone MVC^b^ (N)	−0.36^*^	−0.02	−0.11
Lateral MVC (N)	−0.11	−0.06	−0.02
Motor variability (cm)	−0.27^*^	−0.04	−0.21
NMFslope^c^ (%S^−1^)	0.14	−0.15	0.23^*^
SBST score	0.43^**^	0.12	0.40^**^

Available paired data range from 53 down to 35, due to incomplete set of EMG data. Note that Kendall tau rank correlation coefficients are reported and that they are usually lower than Pearson *r*. Equivalence is determined by the formula *r* ≈ sin(π × τ/2) [38]

^*^ *p* < 0.05; ^**^ *p* < 0.001

^a^STarT Back Screening Tool

^b^Maximal isometric voluntary contraction

^c^Normalized median frequency slope

**Table 3:** Correlation coefficients between both the SBST^a^ and physiologic measures, and outcome variables at follow-ups^b^

Variables	Disability				Pain intensity				Fear of movement			
	T1	T2	T3	T4	T1	T2	T3	T4	T1	T2	T3	T4
Prone MVC^c^ (N)	−0.26^*^	−0.33^**^	−0.16	−0.25	−0.04	−0.13	0.14	−0.10	0.04	0.13	0.32^*^	0.00
Lateral MVC (N)	−0.08	−0.29^*^	−0.12	−0.30^*^	−0.08	−0.25	−0.01	−0.03	0.05	0.07	0.22	0.09
SBST score	0.31^**^	0.27^*^	0.27^*^	0.22^*^	0.17	0.10	0.18	0.34^**^	0.21^*^	0.15	0.18	0.16

Available paired data range from 49 down to 35 participants, due to technical problems and lost to follow-up

^*^ *p* < 0.05; ^**^ *p* ≤ 0.01

^a^STarT Back Screening Tool

^b^2-month (T1), 4-month (T2), 6-month (T3) and 12-month (T4) follow-ups

^c^Maximal isometric voluntary contraction

### Discriminative ability of the SBST and physiologic measures

**Fig. 4:**
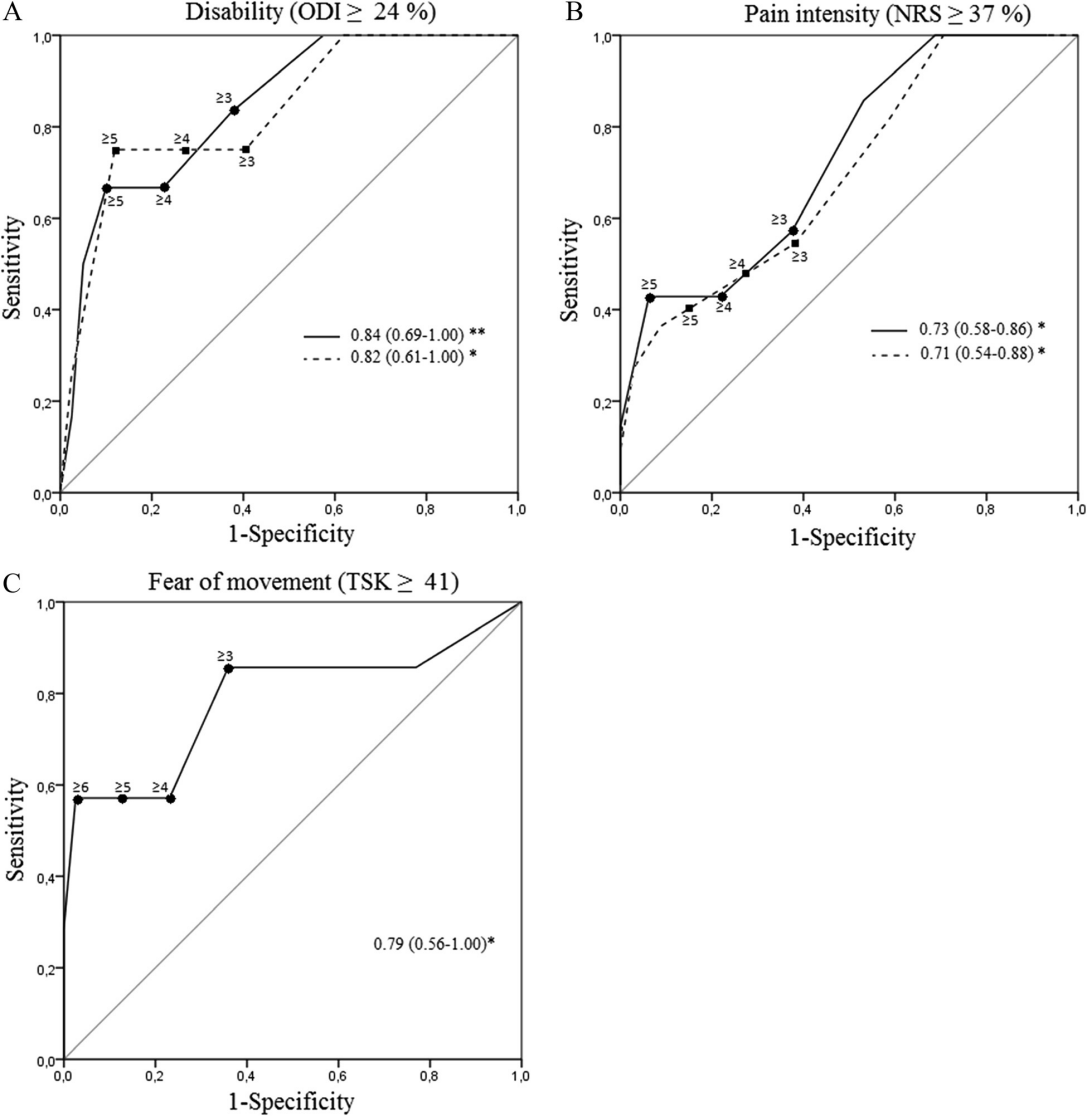
ROC curves at 6- (full line) and 12- (dashed line) month for the SBST against outcome variables. Area under curve (AUC) are reported with 95 % CI in regard of **a**. Disability, **b**. Pain intensity and **c**. Fear of mbovement. * *p* < 0.05 and ** *p* ≤ 0.01

ROC analysis revealed that no physiologic singular-measure had the ability to identify participants presenting higher levels of disability (ODI ≥24 %), pain (NRS ≥37 %), fear of movement (TSK ≥41/68) or absence of subjective status change (PGIC ≥3/7) at T3 or T4. The SBST, however, had an excellent ability to identify participants presenting higher levels of disability at T3 and T4. It also presented an acceptable ability in terms of pain at T3, and T4. Participants presenting higher levels of fear of movement were only significantly identified at T3. The SBST had no ability to identify participants presenting an absence of subjective status change at both T3 and T4. Significant ROC analyses are presented in Fig. 4.

**Fig. 5:**
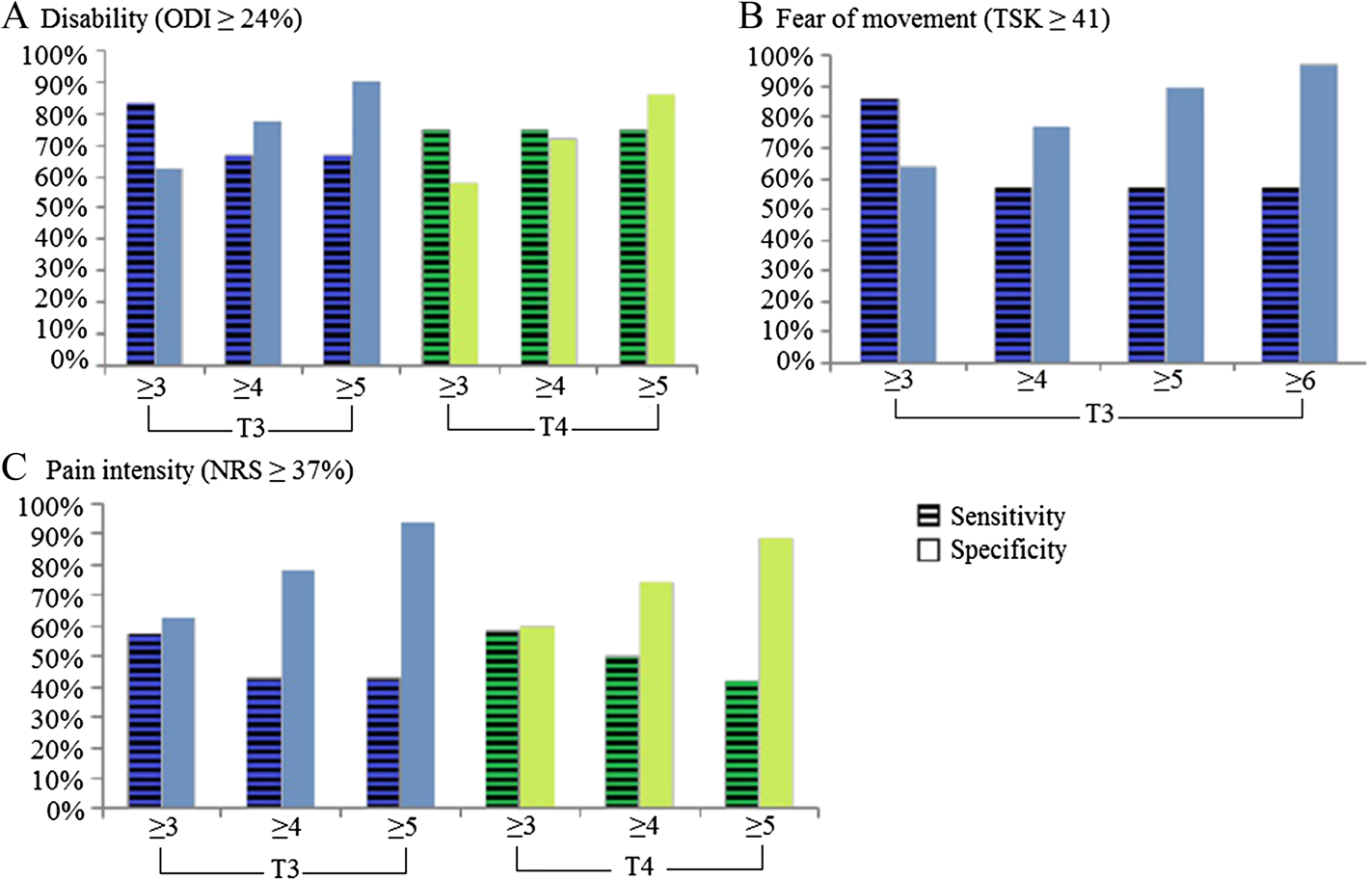
Sensitivity and specificity for STarT Back Screening Tool (SBST) cut-offs. Sensitivity and specificity in the identification of participants presenting higher levels of disability (**a**), fear of movement (**b**), and pain (**c**) at 6- (T3) and 12-month (T4) follow-ups

Since no significant AUC was found for physiologic measures, all other parameters including sensitivity, specificity and positive and negative likelihood ratios (LRs) were only calculated for different SBST cut-offs (see Fig. 5 for sensitivity and specificity values). The cut-off value of ≥ 4, which represents the proposed value to discriminate between low and medium/high risk groups of persistent disabling LBP [13], showed specificity values ranging between 72.1 and 78.1 % and sensitivity values ranging between 42.9 and 75.0 % regarding disability and pain at T3 and T4, and fear of movement at T3. Furthermore, positive and negative LRs for this cut-off were ≤ 2.96 and ≥ 0.35 respectively, depending on both the dichotomous outcomes and the period evaluated. These LRs classify the SBST as a “sometimes useful test” for identifying those presenting disability, pain or fear of movement at 6- and 12-month follow-ups.

## Discussion

The present study was conducted to assess associations between the short- (≤6 months) and long- (12 months) terms clinical status and two types of variables, the SBST and physiologic measures, in patients with chronic LBP. The results showed that, while some physiologic measures are only moderately associated with clinical outcomes over a 12-month period, the SBST questionnaire presents an acceptable or even excellent ability to identify patients presenting higher levels of disability, pain or fear of movement in both the short- and long terms.

### Discriminative ability of physiologic measures

Physiologic measures, such as trunk endurance and MVC, have been reported to be lower in patients with LBP compared to healthy individuals [41, 42]. The present results showed that only MVC obtained in prone or lateral position is related to disability (50 % of the follow-ups) and fear of movement (only at 6-month follow-up). Such results raise concern related to the clinical relevance of physical fitness tests with regard to the evolution of the clinical status in patients with chronic LBP. Since patients with chronic LBP may limit their performance during endurance and MVC tests due to fear of movement or catastrophizing behaviors, some authors have proposed submaximal tests performed in a nearly upright trunk posture [43] or submaximal functional tests [44, 45]. These tests may better reflect the true physical fitness of patients with chronic LBP by the fact that patients do not have to perform maximal efforts. However, the discriminative ability of these tests remains to be investigated and compared to maximal endurance and MVC tests.

### Discriminative ability of the SBST

In contrast to physiologic measures, the SBST allowed for the identification of participants presenting higher levels of disability, pain, or fear of movement at 6 and 12 months (except for fear of movement, which was only significant at short-term). Previous studies reporting the SBST AUC against disability presented results only for baseline analyses [13, 37] or shorter follow-up periods (3-month [14] or 4-month [36]) and involved patients reporting LBP of any duration (acute, subacute or chronic). Because the natural course of acute/subacute and chronic LBP differ, comparisons with previous studies are thus limited [9]. However, regardless of LBP duration, the results of the present study for short-term analyses may be compared to those presented by Morso et al. [14]. The results of the present study showed an excellent ability for the SBST to identify participants presenting higher levels of disability at 6 months, while these authors reported only an acceptable ability to identify patients with a score > 30 on the 101-point Rolland Morris Disability Questionnaire (RMDQ; which is equivalent to a score > 7 on the 25-point RMDQ) at 3-month follow-up. Since an ODI score of 24 has been reported to be equivalent to a RMDQ score of 7 [46], this difference is likely explained by the fact that the Morso et al. study mostly included patients with acute/subacute LBP (63 %). This hypothesis is further supported by Morso et al. [16] who reported a lower predictive ability in a secondary care setting which included 80 % of patients with chronic LBP.

The SBST cut-off between low and medium/high risk groups of persistent disabling LBP has originally been developed in patients presenting LBP of any duration (less than 1 month to more than 3 years duration) [13]. These authors reported sensitivity and specificity indices of 80.1 and 65.4 % respectively in identifying patients with a RMDQ score of ≥ 7 at 6 months. For the same cut-off (i.e. SBST cut-off ≥ 4), the present study showed a lower sensitivity but a higher specificity at both short- (66.7 and 77.5 %) and long- (75.0 and 72.1 %) terms. Because the aim of Hill et al. [13] study was to assess the external validity of the SBST, the authors only presented the sensitivity and specificity of the SBST cut-off ≥ 4 (for the total cohort and for the 5 subgroups based on LBP duration). Nevertheless, it would have been interesting to compare the results of the present study to those of the chronic subgroups of patients to confirm the presence of a different cut-off when only patients with chronic LBP are evaluated.

Since the SBST was first developed and validated with the intent to screen for back pain prognostic indicators relevant to initial decision making in primary care for the entire spectrum of patients with nonspecific LBP, the presence of “outcome dependant cut-offs” raises a concern with regard to which outcome variable should be used to establish a stratified care strategy for chronic LBP. Along with disability, pain, function and quality of life have also been identified has important outcomes by patients with chronic LBP [33] and should perhaps be considered in stratified care approaches. For instance, the absence of significant correlations between the SBST and the PGIC scale may reflect the fact that other health related domains important to patients with cLBP may not be captured by the SBST questionnaire [30].

### Strength and limitations

The main strength of the present study is that it evaluated the associations between the clinical status over a 12-month period and two types of variables, physiologic measures and a brief questionnaire. Furthermore, this study was able to attract a wide range of patients with chronic LBP and maintain a low attrition rate, which increased its ecological value. Most participants reported having being treated for their LBP during the course of the study, which may have affected the outcome measures. Nevertheless, this is consistent with the definition of prognosis factors which refers to generic predictors that are not necessarily unique to a particular intervention [47]. Other limitations include the inability of some participants to properly perform the physical tests (endurance and MVC tasks) and the presence of technical problems with EMG and MVC acquisition, which generated a loss of data. Finally, future studies investigating subgroups of cLBP should consider using the SBST as a categorical variable.

## Conclusion

Although physiologic measures obtained during prone and lateral tests (endurance and MVC) may be used to initially evaluate physical fitness impairment in patients with nonspecific chronic LBP, the results of the present study showed that these tests have limited associations with the clinical status over a 12-month period. On the other hand, the SBST can identify patients presenting higher levels of disability, pain or fear of movement at short- (6-month) and long- (12-month) terms. However, the SBST cut-off score that best identify participants varies depending upon the period and the outcome variable evaluated. Considering the increasing use and usefulness of the SBST, further studies should investigate how various outcomes and subgroups cut-offs could help discriminate between subpopulations of patients with low back pain.

## Abbreviations

AUC: Area under ROC curve
EMG: Electromyography
LBP: Low back pain
LR: Likelihood ratio
MF: Median frequency
MFslope: MF slope
MVC: Maximal isometric voluntary contraction
NMFslope: Normalized MFslope
NRS: Numerical rating scale
ODI: Oswestry disability index
PGIC: Patient’s global impression of change scale
RMDQ: Roland Morris disability questionnaire
RMS: Root mean square
ROC: Receiver operating characteristic
SBST: Subgroup for targeted Treatment (STarT) Back Screening Tool
T0: Baseline assessment
T1 to T4: 2-, 4-, 6- and 12-month follow-up assessments
TSK: Tampa Scale for Kinesiophobia
